# Climate drivers of the 2017 devastating fires in Portugal

**DOI:** 10.1038/s41598-019-50281-2

**Published:** 2019-10-10

**Authors:** Marco Turco, Sonia Jerez, Sofia Augusto, Patricia Tarín-Carrasco, Nuno Ratola, Pedro Jiménez-Guerrero, Ricardo M. Trigo

**Affiliations:** 1 Earth Science Department, Barcelona Supercomputing Center (BSC), 08034 Barcelona, Spain; 2 Regional Atmospheric Modeling (MAR) Group, Department of Physics, University of Murcia, 30100 Murcia, Spain; 3 EPIUnit - Instituto de Saúde Pública, Universidade do Porto, Porto, Portugal; 4 Centre for Ecology, Evolution and Environmental Changes, Faculdade de Ciências, Universidade de Lisboa (CE3C-FC-ULisboa), Lisboa, Portugal; 5 LEPABE-Laboratory for Process Engineering, Environment, Biotechnology and Energy, Faculty of Engineering, University of Porto, Rua Dr. Roberto Frias, 4200-465 Porto, Portugal; 6 Biomedical Research Institute of Murcia (IMIB-Arrixaca), 30120 Murcia, Spain; 7 Instituto Dom Luiz (IDL), Faculdade de Ciências, Universidade de Lisboa, Lisboa, 1749-016 Portugal; 8 Departamento de Meteorologia, Universidade Federal do Rio de Janeiro, 21941-916 Rio de Janeiro, Brazil

**Keywords:** Climate-change impacts, Environmental impact, Natural hazards

## Abstract

A record 500,000 hectares burned in Portugal during the extreme wildfire season of 2017, with more than 120 human lives lost. Here we analyse the climatic factors responsible for the burned area (BA) from June to October series in Portugal for the period 1980–2017. Superposed onto a substantially stationary trend on BA data, strong oscillations on shorter time scales were detected. Here we show that they are significantly affected by the compound effect of summer (June-July-August) drought and high temperature conditions during the fire season. Drought conditions were calculated using the Standardized Precipitation Evapotranspiration Index (SPEI), the Standardized Precipitation Index (SPI) and the Standardized Soil Moisture Index (SSI). Then the extent to which the burned area has diverged from climate-expected trends was assessed. Our results indicate that in the absence of other drivers, climate change would have led to higher BA values. In addition, the 2017 extreme fire season is well captured with the model forced with climate drivers only, suggesting that the extreme fire season of 2017 could be a prelude to future conditions and likewise events. Indeed, the expected further increase of drought and high temperature conditions in forthcoming decades, point at a potential increase of fire risk in this region. The climate-fire model developed in this study could be useful to develop more skilled seasonal predictions capable of anticipating potentially hazardous conditions.

## Introduction

Every year, wildfires impact the lives of many people worldwide. These devastating natural disasters cost billions of Euro in direct and indirect damages. For instance, during the summer of 2018, Australia, Greece, North America, Scandinavia (some even within the Arctic Circle) and the United Kingdom experienced unusually destructive wildfires. On both sides of the Atlantic, 2018 will be remembered by the deadliest fires ever recorded affecting the Mediterranean (Greece) and the USA (California). In July, a series of wildfires close to Athens killed 99 people, the deadliest in Greece history^1^. In October, several fires in California were responsible for more than 85 casualties with the vast majority of these taking place in a single fire event in northern California (Camp Fire) that burned more than 10000 homes in the town of Paradise^2^.

Both the occurrence of and changes in climatic extremes constitute a great concern to fire impacts^3,4^. On the other hand, society exposure to large wildfires has also increased as a result of a significant increase in Wildland-Urban Interface (WUI)^5,6^.

The situation in the Mediterranean basin allows the coexistence of climate patterns typical of sub-tropical and mid-latitude areas^7^ and where both human beings and ecosystems suffer frequently from intense natural hazards, such as droughts^8,9^, heatwaves^10,11^ and wildfires^12,13^ that will likely be more common and intense under climate change^14,15^. Mediterranean ecosystems are prone to forest fires^16^ and a number of extreme fire seasons have struck both the western (2003) and the eastern (2007) Mediterranean and were associated with unusually intense heatwaves^10^. These fires (with an average of approximately 4500 km^2^ burned area every year) caused a massive impact on the economy and the environment, with substantial impacts to carbon sequestration, existence of raw materials, and human casualties^13^.

The year of 2017 was particularly harsh in the Southern Europe, with extensive and powerful wildfires taking place in Portugal, Spain, southern France and Italy, linked with abnormal droughts and heat-waves^17,18^. Besides the impacts on the economy and the environment of those countries, these events also brought a considerable loss of human lives^17^. Portugal, the country with the highest density of ignition and burned area^19^, had an extended and extraordinarily intense fire season with a record total burned area of about 500 000 hectares and more than 120 fatalities in 2017. Two particularly tragic events took place before (17–20 June) and after (15–17 October) the official fire season window established by the Portuguese authorities. According to Sánchez-Benítez *et al*.^18^ the 14–20 June heat-wave event was characterized by an intrusion of warm air resulting from a never before seen subtropical ridge. These authors show that, although the anomalous atmospheric circulation was the key process, the thermo-dynamical changes of the last decades played a decisive role. The largest fires in central Portugal (Pedrógão Grande and Góis) started on June 17th and were characterised by anomalous high temperatures and low relative humidity, but also associated with a very unstable atmosphere that favoured the formation of convective cells and thunderstorms. According to the report from the Portuguese Meteorological Services (IPMA) this instability, further strengthened by local fires, also led to several downburst events and associated gust fronts that enabled the fast spread of the fire^20^. The case of October was marked by strong and persistent southerly winds caused by the close passage of hurricane Ophelia moving northward. Together with dry vegetation and soil due to dry and high temperatures throughout 2017, this meteorological event created the conditions for the extreme fire events of October 15th.

Also, these fires generated an intense smoke plume that travelled long distances, transgressing international boundaries and affecting Northern European countries^21^. Smoke from forest fires is composed of hundreds of chemicals, many of which are known to affect air quality and be harmful to human health^22,23^, and contribute to climate change^24^.

Wildfires are very sensitive to climate variability and changes^3,25^. In particular, fires in western Iberia have been shown to be well related to specific climate variables such as drying conditions and the occurrence of heatwaves during the summer^26,27^. Enhancing the understanding of how climate change and extreme weather events influence the evolution of the burned area is crucial to assess regional vulnerabilities to climate change and may provide the basis to develop adequate adaptation measures. However, predicting fires is complex^28,29^ due to the concomitance and confounding effects of numerous drivers^30–32^ and the difficulty in obtaining field-based observations. In any case, several studies found that summer droughts and high temperatures are primary determinants of the interannual variability of fires in Southern Europe^33–35^.

Given this relationship, we analysed the extreme 2017 fire season in Portugal to identify the drivers responsible for burned area variability in Portugal, unravelling the determinants accountable for gradual and yearly changes. Specifically, the aims of this study are two: (1) assess the magnitude of the variability in burned area related to concurrent high temperature and drought conditions and (2) deliver a quantitative estimate of the effect on fires of the climate trends observed.

## Results

**Figure 1:**
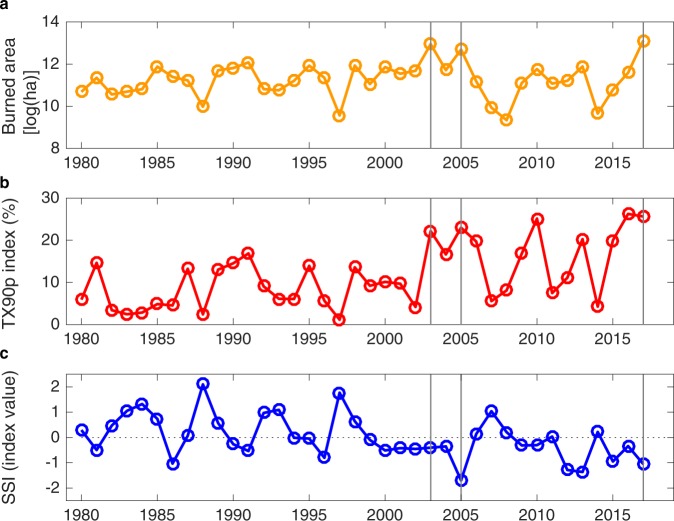
Time series of (**a**) burned area in Portugal over the period from 1980 to 2017 for a June-October fire season and of the summer (June to August) (**b**) percentage of days with maximum daily temperature above the 90th percentile (TX90p index) and of (**c**) drought index based on soil moisture (SSI). Vertical grey lines indicate the top 3 burned area years.

Figure 1a shows the burned area (BA) in Portugal from 1980 to 2017, considering that the fire season comprises the months of June to October. The record-breaking wildfire season of 2017 is evident. However, the BA (log-transformed) time series does not show a clear multi-decadal trend. The Mann-Kendall test confirms that the trend is positive but not significant (*p*-value > 0.05). Superimposed onto this trend, marked year-to-year oscillations are present.

As seen in Fig. 1, these BA fluctuations are related to climate indicators. Two climate indices were considered: the summer (June to August) percentage of days with maximum daily temperature above the 90th percentile (TX90p index; Fig. 1b) and standardized soil moisture index^36^ (SSI; Fig. 1c), defined as soil moisture deficits relative to the climatology. Positive and negative values of the SSI indicate wet and dry conditions, respectively. These periods were chosen to calculate the climate indicators after testing for several time windows (see Methods).

As highlighted in Fig. 1 with grey lines, the top 3 burned area values correspond to summer periods with high temperature extremes (as represented in Fig. 1b with the TX90p index) and dry conditions (i.e. negative SSI values in Fig. 1c). The analysis of (Spearman) correlation values indicates that both TX90p (0.65, *p*-value < 0.01) and SSI (−0.59, *p*-value < 0.01) are related to BA.

Interestingly, while BA does not exhibit any statistically significant trend, TX90p is increasing (4%/decade, *p*-value < 0.01) and SSI is decreasing (−0.4/decade, *p*-value < 0.01), i.e. drought conditions are increasing. However, if temperature and dry conditions are the main driver of fires and shows a progressive increase over the years, why are these trends not reflected into an increase in BA? In order to answer this question, BA long-term changes driven by climatic conditions were investigated, developing and applying climate-BA models and unravelling the drivers that cause gradational and interannual changes. Indeed, to translate the potential impacts of climate trend on BA it is necessary to develop models of BA response. Then, to estimate the impact of temperature and drought trends in BA, the statistical models were used to simulate BA for two scenarios of historical TX90p and SSI: (i) actual TX90p and SSI, (ii) detrended TX90p and detrended SSI. The comparison between (ii) and (i) should provide useful insights on the impact of historical TX90p and SSI trends on BA evolution.

A recent study^34^ showed there is a direct association between the BA provoked by summer fires and the drought situations in the vast majority of Mediterranean Europe during the same summer. The methodology followed here extends this study by exploring the role of high temperature combined with drought indicators to explain summer BA. The likely link of year-to-year (t) changes in BA during summertime with the detrended SSI (i.e. SSI′) and detrended TX90p (i.e. TX90p′) is expressed using the following model:

1 $$\log \,[BA(t)]=\beta 1+\beta 2\cdot SSI^{\prime} (t)+\beta 3\cdot TX90p^{\prime} (t)+\varepsilon (t)$$

where BA(t) stands for the BA in the summer t; *β*1 is the intercept; *β*2 denotes the sensitivity of BA to dry conditions as indicated by the detrended summer SSI; *β*3 is the coefficient of the detrended summer TX90p index; and *ε* is a term that represents the stochastic noise capturing all other factors affecting BA different from SSI′ and TX90p′, including, e.g., forest fire prevention and fire fight efforts. Prior to the analysis, the predictors are standardized (see Methods). This standardization makes the coefficients of regression model comparable with each other. Since BA follows approximately a log-normal distribution, the variable has been normalized by a log transformation. The resulting model is:

2 $$\log \,[BA(t)]=11.26-0.32\cdot SSI^{\prime} (t)+0.45\cdot TX90p^{\prime} (t)$$

Importantly, the variance explained for the log(BA) model is 0.61, pointing out the skilful performance of this parsimonious model (that includes only two predictors) to reproduce the observed BA. Besides, the coefficients of the model are statistically significant (*p*-value < 0.01), with bootstrapped 95% confidence intervals spanning between (−0.46 and −0.16) for the *SSI*′ coefficient, and between 0.24 and 0.67 for the *TX90p*′ coefficient. These key variables show significant correlation (−0.45, *p*-value < 0.01), indicating that (i) dry summers are usually hot in this area and (ii) that there is the danger of over-fitting in the regression model. To demonstrate the importance of considering both variables, two simpler models were tested, based on *SSI*′ or on *TX90p*′ alone. Both models show lower explained variance (R^2^), with the *SSI*′ model yielding a R^2^ value of 0.39, while the R^2^ value of *TX90p*′ model is 0.50. Also, the Akaike Information Criterion score has been calculated (*AIC*, adjusted for finite sample size). AIC is a score usually used on statistical models to quantify their skill, and is substantiated on a trade-off between complexity (that is, the number of free parameters) and its accuracy (explained variance). The application of this criterion permits to select that model with the lowest AIC score. Henceforth, this analysis settles that the regression model including both variables has the largest explanatory power with the highest simplicity, with an AIC of −29 considerably better than the model based solely on *SSI*′, with an AIC of −14, or the model based on *TX90p*′, with an AIC of −22.

To support the selection of SSI as the best drought predictor for BA, we repeat the analysis using other indicators including the Standardized Precipitation Index (SPI; which is mathematically similar to SSI, but is based only on precipitation) and the Standard Precipitation and Evaporation index (SPEI). Table 1 summarises the results for all indicators. The three models perform similarly, with best results using the SSI index. Interestingly, while the SPI index does not show any significant trend, the SPEI displays a negative trend (−0.3, *p*-value < 0.05), in line with the SSI trend.

**Table 1:** Model results considering three different drought indicators: the Standardized Precipitation Index (SPI), Standard Precipitation and Evaporation index (SPEI) and the Standard Soil moisture index (SSI).

drought indicator	Model	Explained Variance
SPI	log[*BA*(*t*)] = *11*.*26* − *0*.*26* · *SPI*′(*t*) + 0.53 · *TX*90*p*′(*t*)	0.59
SPEI	log[*BA*(*t*)] = *11*.*26* − *0*.*30* · *SPEI*′(*t*) + 0.40 · *TX*90*p*′(*t*)	0.57
SSI	log[*BA*(*t*)] = *11*.*26* − *0*.*32* · *SSI*′(*t*) + 0.45 · *TX*90*p*′(*t*)	0.61

Henceforth, drought and high summer temperatures are both significant predictors for BA, and the omission of one of these two variables could conduce to a reduced ability to explain fire variations. Also, the climatic variables used by the empirical model allow the coefficients in Eq. 2 to provide estimations of the relative weight of the diverse climate predictions, since the variables are regional means of standardized series (dimensionless). The weight of the extreme temperature indicator is larger than that of SSI′, indicating that for this region, high temperature values seem to drive BA fluctuations more effectively than drought.

It is always necessary to validate the quality and robustness of statistical model to perform out-of-sample prediction^37^. This prediction implies using one subset of the data (training set) to determine the model parameters, while a second subset (testing set) is employed to validate the prediction. In our approach, we have applied a leave-one-out cross-validation method that consists on a one-year moving window, while the rest of the observations are the training data. Noticeably, the difficulty to obtain a good out-of-sample prediction is higher than of a pure hindcast (reproduction), particularly if there is a time-limited calibration period. The methodology of Calmanti *et al*. (2007; see Methods)^38^ has been followed in order to estimate the uncertainty of the prediction.

**Figure 2:**
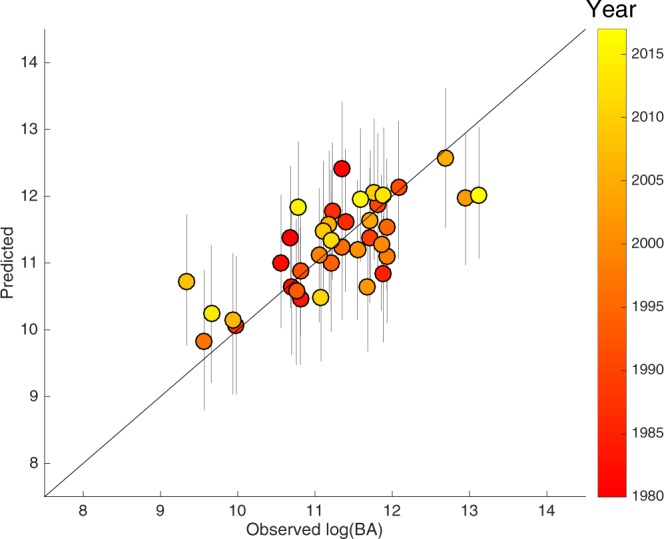
Observed log-transformed burned area (ha) against out-of-sample predictions. The year for each value is depicted by the coloured points. Grey vertical bars enclose 95% confidence intervals of the predictions. Data corresponds to the extended fire season (June-October) burned area (BA) for Portugal between 1980 and 2017.

Figure 2 shows the observed vs modelled log(BA) including the uncertainty bands. An ensemble of the 1000 out-of-sample predictions has been used to demarcate the uncertainty bands, defined by the 2.5th and the 97.5th percentiles. The correlation of the data with the out-of-sample predictions (r = 0.73, *p*-value < 0.01) indicates an accurate model performance also in prediction mode. The summer years are shown with different colours in Fig. 2, suggesting that the relationship has not varied substantially and can be considered stationary in time.

As shown in the analysis, this simple regression model provides skilful out-of-sample predictions of the influence of climate variability on summer fires, suggesting that it may be utilized to assess the impact of observed temperature trends on BA. Hence, while previously the year-to-year changes in the response BA variables we have modelled using detrended SSI and TX90p, we now estimate BA changes considering the non-detrended climate indicators. This approach utilized a methodology for the trend attribution extensively used to examine the impacts of climate change on crops (*e*.*g*. Lobell^39,40^) and has been previously applied to analyse the observed climate change impact of fire in Catalonia (NE of Spain^41^) and in southern France^42^. This type of model also assumes that the response of fires is analogous for inter-annual climate fluctuations and for longer-term climatic trends.

**Figure 3:**
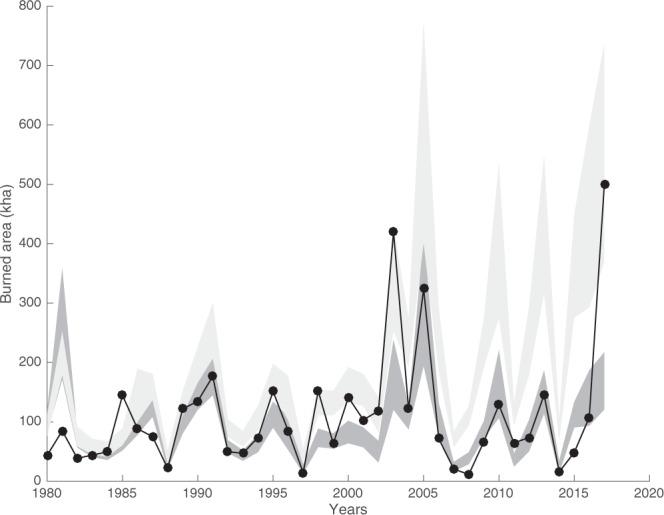
Deviations between climate-driven trend of summer burned area (light grey) and observed values (black line with solid circles). The dark grey band shows the model results considering the detrended drought and temperature indices (Eq. 2), while the light grey shaded band refers to non-detrended series (Eq. 3). The shaded bands include 95% of the members of 1000 different bootstrap replicates.

Figure 3 depicts the output of the multi-linear model by means of SSI and TX90p or detrended SSI and TX90p. The results highlight the evident impact of climate change on BA. Whilst the real trend of BA is not significant, a positive trend in BA would have been produced by climate forcing alone. It should also be emphasized that only the model including the original (i.e. non detrended) SSI and TX90p series is able to capture the extreme 2017 fire season.

## Discussion

Our study covering the last 38 years (1980–2017) reveals that both high temperature and drought (by the SSI index) have been important drivers for the BA in Portugal. The solid association of BA with these factors ascribe that the process by which climate influences BA is, overall, straightforward: drier and warmer conditions result in larger wildfires. The same result was found in other studies, where drought and maximum temperatures during the fire season have frequently been linked with fire activity in the Mediterranean and in other regions of the world^33,43–46^.

However, while the temperature and the soil moisture drought conditions in the previous decades have increased, the measured trend of BA is substantially stationary. Our results also reveal that, in fact, in the absence of other drivers, climate change would have led to higher BA values. The 2017 extreme fire season is, however, an anomaly in this context, as only the climate-fire model that includes merely climatic drivers is able to capture the observed value.

The BA is consequence of several factors, notably: biomass (potential fuel), availableness to burn (dictated by moisture, which is regulated by recent weather and drought) and the probability of fire spread in response to weather conditions^47^. The second and third factors depend totally on climate variables, which cannot be controlled; actually, over the last decades these have been moving towards warmer and drier summers^48^. Despite the improvement of the fire management efforts tried in the last years, some difficulties are reported to incorporate best administration policies and their implementation, risk-based planning, and funding instruments simultaneously in a long-term planning scheme^19^. That is, implementing risk-based planning in a long- term planning scheme would improve fire management efforts.

In relation to biomass, Portugal has seen a reduction of its forest surface over time, with losses of 4.6% (approximately 104 ha per year) between 1995 and 2010^49^. This reduction was in part due to a decrease in the area covered by maritime pine (*Pinus pinaster*). Wildfires, together with slash of trees to control the spread of the pine wilt nematode, were the main factors behind this decrease^19^. Fire return intervals of <20 years, just like in Portugal, do not allow this pine species to reach maturity and produce seeds, which compromises its regeneration^50^. The forest surface covered by eucalyptus (*Eucalyptus globulus*), on the other hand, has slightly increased (though not enough to counterbalance the decrease of pine area). The proliferation of eucalyptus is in part explained by the wildfire occurrence, which encouraged forest owners to replace pine by species with shorter life-cycles, and therefore compatible with recurrent fires^26^. The reduction in the forest area could have had a role in the steady BA values over time.

Notwithstanding human and fires have been living together for a long time^51^, our capability to manage fire remains restricted and may become even more complicated in the future^30,52^ due to climate, vegetation and fire regime changes. Improving the knowledge on the main drivers of the year-to-year variation of fire at regional scale, is fundamental to better understand fires and predict their change, as well as to deliver new information for management purposes. For instance, the established climate-fire model in this study could be useful to develop more skilled seasonal predictions capable of anticipating potentially hazardous conditions^53^. Taken together, our results enhance the knowledge on the impact of climate change on wildfires in Mediterranean areas, delivering a climate change-fire model that can be adapted to be used in other geographical regions.

## Methods

### Climate and fire data

Monthly Burned Area (BA) data for Portugal for the months between June and October, were obtained from the EFFIS^54^ for the period 1980–2015 and from the Nature Conservation Institute and Forestry of Portugal for 2016 and 2017 (www2.icnf.pt/portal/florestas/dfci/inc/info-geo).

The gridded meteorological dataset E−OBS (version 19.0^55^) was used at 0.1° spatial resolution to compute the climate indicators.

The monthly TX90p indicator was calculated as the percentage of daily maximum temperature above the 90th percentile. This threshold is calculated for each calendar day using a 5-day window, and a bootstrap procedure^56^ to avoid inhomogeneity at the beginning and end of the period used to calculate the percentile (here 1981–2010).

We consider three different drought indicators: the Standardized Precipitation Index (SPI^57^), the Standard Precipitation and Evaporation index (SPEI^58^), and the Standardized Soil moisture Index (SSI^36^). SPI is a transformation of the accumulated precipitation values over a specific period (e.g. 3 months) into a standard Gaussian distribution with mean 0 and standard deviation 1. Positive values indicate surplus of rainfall, whereas negative values identify dry conditions relative to the long-term climatology. The SPEI indicator is mathematically similar to SPI, but also includes the effects of temperature, that expresses the potential evaporation^58^. SPEI uses as an input a water balance, considering the total accumulated precipitation and PET. The Hargreaves PET estimation method has been considered, taking into account temperature and precipitation in its formulation (plus the latitudinal correction factor). Also, the SSI indicator is mathematically analogue to SPI and could be considered a proxy for agricultural drought, since it is based on anomalies in soil moisture^36^. We calculated the SSI from the soil moisture data obtained through the Copernicus dataset “Essential Climate Variables for assessment of climate variability from 1979 to present” and available here: https://cds.climate.copernicus.eu/cdsapp#!/dataset/ecv-for-climate-change?tab=overview. See also Albergel *et al*.^59^ for a quality assessment of this data. For SPI, SPEI and SSI, the standardization step is based on a nonparametric approach in which the probability distributions of the data samples are empirically estimated^36,60^.

All the temperature and drought indices were calculated for each point of the grid and then spatially averaged over Portugal.

### Statistical analysis

To identify the best model parameters, we fitted all the possible versions of Eq. (1) considering all the possible temporal aggregations of the predictors. Specifically, monthly TX90p data were aggregated in multi-month series called TX90p(a-b), where a is the first month while b is the last (excluding months following October, the end of the fire season considered here), while multi-month drought series are called SSI_t_(m), where m is the month when SSI is calculated (ranging from 1 to 10) and t is the accumulation period (we test here 3, 6 and 12 months), resulting in 1650 possible combinations. We then fitted all these models -considering both regression with the individual predictors (i.e. only TX90p or SSI data) and the multi regression that uses TX90p and SSI- and calculated their AIC and the significance of their (Pearson) correlations through a one-tailed hypothesis test. Then we corrected individual significance tests for multiple hypotheses testing using the False Discovery Rate (FDR) method^61^. Finally, to identify the best model we seeked the minimum AIC value among all the significant models calculated in the previous step. We repeated the same analysis also considering the drought indicators SPI and SPEI.

To estimate the uncertainty of the prediction, the methodology proposed by Calmanti^38^ was followed. The practical implementation of this method is summarized in the following steps:

- The variance of the residuals in the calibration period is estimated;
- Then, an ensemble of 1000 Gaussian, temporally uncorrelated stochastic residual time series are generated, with variance equal to that estimated from the calibration period;
- Finally, the stochastic residuals are added to the predicted model values, generating an ensemble of 1000 predictions, which include the residual stochasticity.

The significance of long-term trends has been assessed by the Mann-Kendall test.
